# Improved HIV Awareness and Perceived Empowerment to Negotiate Safe Sex among Married Women in Ethiopia between 2005 and 2011

**DOI:** 10.1371/journal.pone.0115453

**Published:** 2014-12-15

**Authors:** Zaake De Coninck, Ibrahim A. Feyissa, Anna Mia Ekström, Gaetano Marrone

**Affiliations:** 1 Department of Public Health Sciences/Global Health (IHCAR), Karolinska Institutet, Stockholm, Sweden; 2 CDC/WHO Stop Transmission of Polio/STOP Consultant, Expanded Program of Immunization Unit, World Health Organization, Kenya; 3 Department of Infectious Diseases, Karolinska University Hospital, Stockholm, Sweden; University of Liverpool, United Kingdom

## Abstract

**Introduction:**

The HIV prevalence rate in Ethiopia for married (or cohabiting) women is 3 times that found amongst women who have never been married. While marriage used to be seen as a protective factor against HIV, evidence suggests that this is no longer necessarily the case. This study analyses the trend and socio-demographic determinants of HIV awareness and safe sex negotiation among married women in Ethiopia between 2005 and 2011.

**Methods:**

Data from Ethiopian Demographic and Health Surveys conducted in 2005 and in 2011 were analysed. Socio-demographic variables as well as ‘survey year’ were selected to assess their interaction with selected HIV awareness and safe sex negotiation indicators. Multivariable regression analyses were performed. Odds ratios and confidence intervals were computed.

**Results:**

A significant increase in knowledge of HIV and ability to negotiate safer sex occurred between 2005 and 2011 reflecting a positive trend in gender empowerment amongst married Ethiopian women. Some of these advancements were striking, for instance respondents were 3.6 times more likely to have “Heard of AIDS” in 2011 than in 2005. HIV awareness and safer sex negotiation were significantly associated with higher education, higher socioeconomic status, those who had heard of HIV, those of the Orthodox Christian faith, and (to some extent) those living in rural areas.

**Conclusion:**

HIV awareness has increased significantly in Ethiopia over the last decade but married women are still disproportionately susceptible to HIV. Community programmes, already effective in Ethiopia, also need to target this vulnerable sub-group of women.

## Introduction

At least 1.3 million people are estimated to be living with HIV in Ethiopia, one of Africa's largest countries and home to almost 92 million inhabitants [1]. When the rate of heterosexual transmission was last surveyed in 2005, it accounted for 87% of the cases [2], and as of 2011 the HIV prevalence rate among women was almost twice (1.9%) that among men (0.9%) [3]. In addition, only 35% of Ethiopian women compared to 56% of men understood two of the main ways to prevent HIV transmission (by limiting sexual intercourse to one uninfected partner and by using a condom consistently) [3]. Public health programmes have thus targeted women in order to reduce the country's HIV incidence and prevalence rates.

A vast majority of these programmes focus on premarital relationships – specifically young and never-married individuals [3]. In 1998, the Ethiopian National HIV/AIDS Policy was implemented for over a decade and it highlighted youth as a high-risk group - along with commercial sex workers and their clients, mobile groups (long distance truck drivers, military personnel), street children, refugees, and prisoners [4]. Unlike young or pre-married demographics, older or married women were not recognised as a vulnerable target group. Indeed, the policy highlights the important role to be played by the Ministry of Education: “to ensure that appropriate curriculum and teaching materials shall be developed and implemented for HIV/AIDS/STD in school health education at all levels” and for interventions to be “developed and implemented for youth out-of-school in rural and urban areas” [4]. Ethiopia's Strategic Plan for Intensifying Multisectoral HIV and AIDS Response in Ethiopia II (2009-2014) also highlights never-married sexually active females and youth as some of the most at-risk groups in Ethiopia with regards to HIV. Married and older women are still not a targeted group [5].

These, both previous and current programmes ignore the fact that a significant amount of HIV infection occurs within the context of marital relationships. While marriage used to be seen as a protective factor against HIV, recent findings suggest that this is no longer necessarily the case. Research conducted in Zambia and Rwanda [6], and in Ghana and Kenya [7] concludes that married women are more vulnerable to HIV than non-married women. In South Africa, a population-based study demonstrates that condom use increased amongst casual relationships but not amongst regular partners or married couples [8]. In Indonesia, one study highlights the fact that married women have, in general, less knowledge about HIV transmission than married men and that this was significantly associated with a lack in negotiating sex [9]. Separate studies conducted in Zimbabwe, Uganda, and India show that, despite understanding that they are at an increased risk of contracting HIV, married women face multiple challenges when negotiating condom use with their partner. The reasons for this can be attributed to their economic dependence, gender roles, and the power dynamics women grapple within the relevant societal contexts [10]–[14]. A study conducted in Kenya and in Zambia attributes the heightened risk of HIV to the fact that women tend to marry younger, often to older partners [15] – who will have had increased exposure to sex and HIV/STIs.

In Ethiopia, all of the aforementioned causes (childhood marriage, age disparity between husband and wife, gender roles, and economic dependency) have been recognised as limiting factors for women with regards to negotiating safer sex [16]. Married Ethiopian men in 2011 were considerably more likely to have 2 or more partners in the past 12 months (5.1%) when compared to non-married men (1.2%) or women (0.3%) – regardless of whether they were married or not [3]. Furthermore, married women scored lower when it came to negotiating safer sex than non-married women: married women were less likely than non-married women to feel justified in refusing to have sexual intercourse if she knew her husband had sex with other women (81% vs. 85%) or even in asking her husband to use a condom if she knew he had an STI (65% vs. 78%) [3]. The trends between 2005 and 2011 also largely suggest that more progress has been accomplished amongst never-married respondents than amongst married or cohabiting women [2], [3]. For instance, the proportion of women agreeing that they can refuse to have sexual intercourse with her husband if she knows he had sex with other women increased more amongst never married respondents (82% to 85%) than amongst married or cohabiting respondents (80% to 81%). As a result, 2011 estimates place the HIV prevalence rate for married (or cohabiting) women at 3 times (1.5%) higher than the rate observed in never married women (0.5%) although this figure is not adjusted for age and married women are generally older than non-married [3] – 2005 rates were recorded at 1.6% and 0.7% [2]. This statistic is particularly arresting in light of the fact that only one quarter of all women respondents of the 2011 EDHS – a representative sample of the country's demography – had never been married [3].

In order to effectively target relevant national HIV programmes, it is vital to understand the determinants associated with HIV awareness and perceived empowerment to negotiate safer sex (hereafter simply referred to as ‘safe sex negotiation’) amongst married women in Ethiopia. This study analyses the trend and socio-demographic determinants of HIV awareness and safe sex negotiation among married women in Ethiopia between 2005 and 2011 using data from the two most recent EDHS's.

## Methods

### Study setting and data collection

The World Bank estimates that the population of Ethiopia is almost 92 million inhabitants and it has a population growth rate of about 2%. Between 2005 and 2011, the proportion of the rural population remained fairly constant at about 84%, GDP per capita increased from 166 US dollars to 374 US dollars, and life expectancy increased from 55 years in 2005 to 58 in 2009 [1]. Total fertility rate increased from 5.3 to 6 children born per woman [17]. The number of HIV Counseling and Testing (HCT) sites has expanded rapidly: from just 658 in 2004/5 to 2,309 in 2010/11. This expansion has led to a steep rise in the number of people who received HIV tests from 5.8 million in 2009/10 to a record 9.4 million in 2010/11 [18]. The number of Health facilities that provide PMTCT (Prevention of Mother to Child Transmission) has grown from 32 in 2003/4 to 408 in 2006/7 and to 1352 in 2009/10. However, the number of health facilities that provide PMTCT is still below 50% of the total number of health facilities that can potentially provide the service [19].

Demographic and Health Surveys (DHS) are conducted every few years in select low- and middle-income countries with the primary objective of providing data for monitoring and evaluation and policy development purposes [3]. The 2011 and 2005 EDHS were carried out under the aegis of the Ethiopian Ministry of Health and was implemented by the Central Statistical Agency. Assistance to the project was provided through the MEASURE DHS project, a project providing support and technical assistance in the implementation of population and health surveys in countries worldwide. For each survey, representative samples were selected using a stratified, two-stage cluster design. The first stage involved selecting clusters from a list of enumeration areas: 540 clusters were selected in 2005 and 624 clusters were selected in 2011. The second stage involved selecting a list of representative households from each cluster. 13,721 households were selected in 2005 and 17,817 households were selected in 2011. This study makes use of data collected on 8,438 married women out of 14,070 in 2005 and 9,478 married women out of 16,515 in 2011.All respondents were aged 15–49 [2], [3].

### Ethical clearance

DHS data is collected with the respondents' informed consent and is then made publicly accessible. Analysing DHS data from any country, including Ethiopia, does not require additional ethical clearance. All DHS datasets are free to download and to analyse once you complete a short registration (which is free and available for all) with the Demographic and Health Surveys Program.

### Data analysis

**Figure 1:**
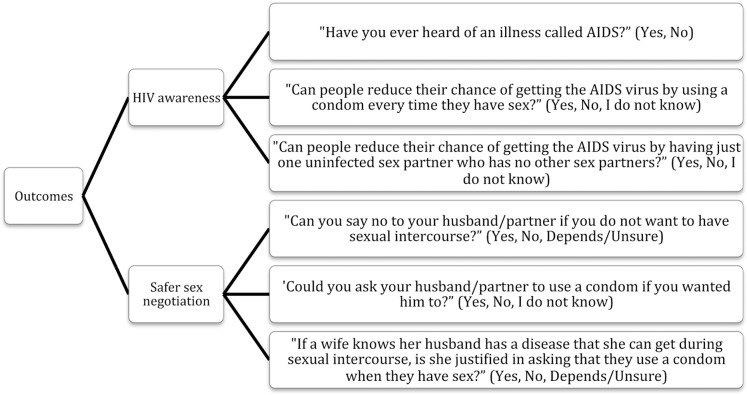
Selected indicators.

Data was analysed using SPSS-PASW, version 18 (SPSS, Inc., Chicago, IL) and STATA version 12.0 (College Station, StataCorp LP, TX, USA). The indicators selected are presented in Fig. 1. The following independent variables were selected to study their relationship with the aforementioned indicators: ‘Year’ (2005; 2011), ‘Age in 5-year groups’ (15–19; 20–24; 25–29; 30–34; 35–39; 40–44; 45–49), ‘Place of residence’ (Urban; Rural), ‘Education level’ (No education; Primary education; Secondary education; Higher education), ‘Working status’ (Yes; No), ‘Economic status’ (Poorest; Poorer; Middle; Richer; Richest), ‘Husband's age in 5-year groups’ (15–19; 20–24; 25–29; 30–34; 35–39; 40–44; 45–49; 50+), ‘Husband is older/younger’ (Younger; Same age; Older), ‘Husband's education level’ (No education; Primary education; Secondary education; Higher education; Don't know), ‘Respondent is a mother’ (No; Yes), ‘Religion’ (Orthodox Christian; Christian - Catholic & Protestant; Muslim; Other). Finally, when studying the indicators pertaining to ‘Safer sex negotiation’ (‘Can refuse sexual intercourse’, ‘Can ask husband to use condom anytime she wants’, ‘Justified to ask husband to use condom if he has an STD’), the variables “Ever heard about HIV/AIDS” and “Ever heard about STIs” were listed as independent variables to study the hypothesis that greater knowledge about HIV/STI empowers negotiation.

The association between each indicator and each independent categorical variable was first assessed using Chi-square or Fisher's exact test. The independent variables with a p-value equal to or less than 0.20 were then entered into binary (for binary outcomes) or multinomial (for outcomes with three categories) logistic regression models. When multinomial logistic regression models were created, only the Odds Ratios (OR) and their 95% Confidence Interval (CI) for women answering “Yes” versus “No” have been presented, ignoring the parameters for “I do not know” versus “No”, and “Depends/Unsure” versus “No”. This was done to simplify the presentation and interpretation of data. For the same reasons, only the significant variables have been presented. Both backward and forward stepwise logistic regression analyses (Wald test) with a p-value cut-off of 0.20 were performed and they gave the same results. P-values less than 0.05 (two sided test) were considered significant in the final model.

## Results

**Figure 2:**
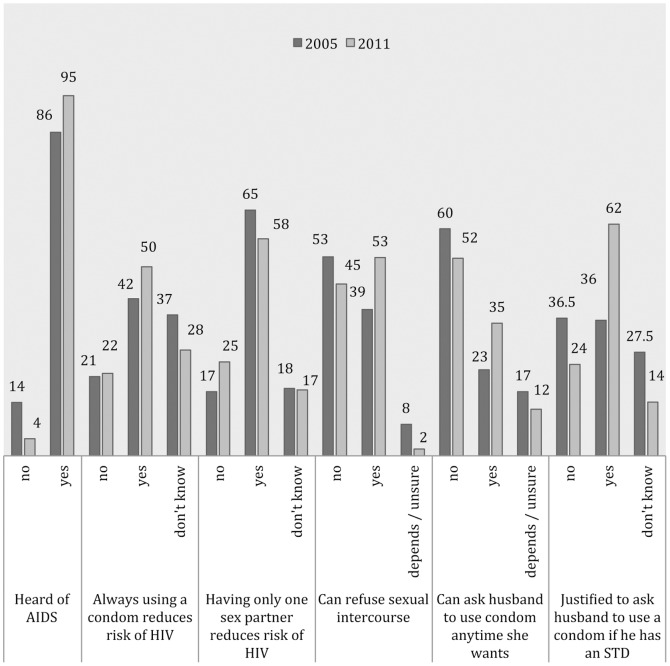
Frequency distribution of HIV awareness and perception to negotiate safer sex (%).

Table 1 reflects the positive trend in urbanisation, level of education (for both respondents and their husbands), and level of employment over time – even if education (66% for respondents) and employment levels (69%) remained low in 2011. Most respondents were either Muslim or Orthodox Christian, followed by Christian (Catholic &Protestant), then believers of ‘Other’ faiths. The distribution of socioeconomic status, the proportion of respondents that were mothers (about 90%), and the proportion of women that married older men (over 85%) remained fairly evenly distributed within and between surveys. Fig. 2 highlights an overall improvement in HIV awareness and empowerment to negotiate safe sex between 2005 and 2011 although there was a decrease in proportion of respondents agreeing that “Having only one sex partner reduces risk of HIV” (from 65% down to 58%).

**Table 1:** Socio-demographic characteristics of the 8,438 respondents participating in the 2005 survey and the 9,478 respondents participating in the 2011 survey surveys.

Socio-Demographic Characteristics		2005 (N = 8,438)		2011 (N = 9,478)	
		N	%	N	%
Age (years)	15–19	701	8.3	720	7.6
	20–24	1,444	17.1	1,617	17.1
	25–29	1,938	23.0	2,299	24.3
	30–34	1,424	16.9	1,597	16.8
	35–39	1,285	15.2	1,511	15.9
	40–44	878	10.4	986	10.4
	45–49	768	9.1	748	7.9
Residence	Urban	1,626	19.3	2,093	22.1
	Rural	6,812	80.7	7,385	77.9
Education	No education	6,279	74.4	6,266	66.1
	Primary education	1,283	15.2	2,433	25.7
	Secondary education	739	8.8	462	4.9
	Higher education	137	1.6	317	3.3
Economic status	Poorest	2,015	23.9	2,613	27.6
	Poorer	1,471	17.4	1,586	16.7
	Middle	1,421	16.8	1,493	15.7
	Richer	1,352	16.0	1,521	16.0
	Richest	2,179	25.8	2,265	23.9
Currently working	No	6,339	75.1	6,450	68.1
	Yes	2,098	24.9	3,014	31.9
Religion	Orthodox Christian	3,601	42.7	3,415	36.0
	Christian (Catholic & Protestant)	1,457	17.3	1,715	18.1
	Muslim	3,170	37.6	4,190	44.2
	Other	206	2.4	154	1.6
Husband's age (years)	15–19	38	0.4	32	0.3
	20–24	507	6.0	518	5.5
	25–29	1,189	14.1	1,378	14.5
	30–34	1,431	17.0	1,695	17.9
	35–39	1,411	16.7	1,616	17.0
	40–44	1,169	13.8	1,457	15.4
	45–49	962	11.4	948	10.0
	50+	1,731	20.5	1,834	19.3
Husband's education	No education	4,921	58.5	4,802	50.7
	Primary education	2,034	24.2	3,259	34.4
	Secondary education	1,164	13.8	739	7.8
	Higher education	282	3.3	573	6.1
	Don't know	16	0.2	90	0.9
Respondent is a mother	No	837	9.9	955	10.1
	Yes	7,601	90.1	8,523	89.9
Husband is older/younger	Younger	103	1.2	151	1.6
	Same age	953	11.3	1,200	12.7
	Older	7,382	87.5	8,127	85.7

Having adjusted for the socio-demographic factors several results stand out regarding HIV awareness (Table 2). There was a statistically significant improvement for 2 of the 3 outcomes over time: for “Ever heard about HIV/AIDS” and for “Always using a condom reduces risk of HIV”, respondents were 3.6 times (OR 3.58, 95%, CI 3.17–4.04) and 33% (OR 1.33, 95%, CI 1.22–1.45) more likely to agree with the statement in 2011. Education and socioeconomic status were some of the variables most strongly associated with HIV awareness. Women currently working were about 20% more likely to agree that “Always using a condom reduces the risk of HIV” (OR 1.22, 95%, CI 1.11–1.34) and that “Having only one sex partner reduces the risk of HIV” (OR 1.19 1.08–1.30). Likewise, Orthodox Christian respondents had higher HIV awareness than respondents of other faiths. A husband's higher education level was inconsistently associated with two of the three indicators. A respondent was more likely to have “Heard of AIDS” as she got older, and if she was the same age or older than her husband. However, a respondent's age and her husband's relative age was not associated with any of the other indicators. None of the HIV awareness indicators were significantly associated with whether a respondent was a mother.

**Table 2:** Logistic and multinomial regression models showing factors significantly associated with knowledge of transmission of HIV.

Socio-Demographic Characteristics		Respondents N (%)	Ever heard of AIDSα	Always using a condom reduces the risk of HIVβ	Having only one sex partner reduces the risk of HIV γ
			OR (95% CI)		
**Year**	2005	8,438 (47.1%)	ref.	ref.	ref.
	2011	9,478 (52.9%)	3.58 (3.17–4.04)***	1.33 (1.22–1.45)***	0.63 (0.58–0.68)***
**Age (years)**	15–19	1,421 (7.9%)	ref.		
	20–24	3,061 (17.1%)	1.48 (1.16–1.89)**		
	25–29	4,237 (23.6%)	1.52 (1.15–1.99)**		
	30–34	3,021 (16.9%)	1.71 (1.25–2.35)**		
	35–39	2,796 (15.6%)	2.02 (1.43–2.85)***		
	40–44	1,864 (10.4%)	2.10 (1.44–3.07)***		
	45–49	1,516 (8.5%)	2.51 (1.68–3.76)***		
**Residence**	Urban	3,719 (20.8%)		ref.	
	Rural	14,197 (79.2%)		0.73 (0.62–0.86)***	
**Education**	No education	12,545 (70.0%)	ref.	ref.	ref.
	Primary	3,716 (20.7%)	2.28 (1.85–2.81)***	1.33 (1.19–1.48)***	1.29 (1.15–1.43)***
	Secondary	1,201 (6.7%)	9.14 (3.63–22.99)***	2.56 (2.04–3.22)***	1.51 (1.23–1.86)***
	Higher	454 (2.5%)	9.49 (1.23–73.48)*	1.90 (1.33–2.70)***	1.32 (0.97–1.79)
**Economic status**	Poorest	4,628 (25.8%)	ref.	ref.	ref.
	Poorer	3,057 (17.1%)	1.53 (1.32–1.78)***	1.38 (1.21–1.58)***	1.35 (1.19–1.53)***
	Middle	2,914 (16.3%)	1.51 (1.29–1.77)***	1.27 (1.11–1.45)**	1.26 (1.11–1.43)***
	Richer	2,873 (16.0%)	1.86 (1.56–2.21)***	1.26 (1.10–1.45)**	1.25 (1.10–1.42)**
	Richest	4,444 (24.8%)	3.26 (2.44–4.35)***	1.59 (1.34–1.89)***	1.37 (1.20–1.57)***
**Working**	No	12,789 (71.4%)		ref.	ref.
	Yes	5,112 (28.6%)		1.22 (1.11–1.34)***	1.19 (1.08–1.30)***
**Religion**	Orthodox Christian	7,016 (39.2%)	ref.	ref.	ref.
	Muslim	7,360 (41.1%)	0.45 (0.39–0.51)***	0.50 (0.45–0.55)***	0.54 (0.49–0.59)***
	Other	360 (2.0%)	0.20 (0.15–0.27)***	0.72 (0.51–1.02)	0.69 (0.50–0.96)*
	Christian (Catholic & Protestant)	3,172 (17.7%)	0.43 (0.36–0.51)***	0.57 (0.50–0.64)***	0.57 (0.51–0.64)***
**Husband's age (years)**	15–19	70 (0.4%)			ref.
	20–24	1,025 (5.7%)			1.77 (1–3.14)
	25–29	2,567 (14.3%)			2.07 (1.17–3.66)*
	30–34	3,126 (17.4%)			2.14 (1.20–3.82)*
	35–39	3,027 (16.9%)			1.91 (1.07–3.42)*
	40–44	2,626 (14.7%)			1.63 (0.90–2.92)
	45–49	1,910 (10.7%)			1.85 (1.02–3.35)*
	50+	3,565 (19.9%)			1.91 (1.06–3.46)*
**Husband's education**	No education	9,723 (54.4%)	ref.		ref.
	Primary	5,293 (29.6%)	1.58 (1.36–1.84)***		1.15 (1.04–1.27)**
	Secondary	1,903 (10.6%)	1.65 (1.21–2.23)**		1.18 (0.99–1.39)
	Higher	855 (4.8%)	1.45 (0.68–3.12)		0.98 (0.77–1.24)
	Don't know	106 (0.6%)	1.83 (0.66–5.07)		0.98 (0.60–1.59)
**Husband is older/younger**	Younger	254 (1.4%)	ref.		
	Same age	2,153 (12.0%)	2.20 (1.41–3.42)***		
	Older	15,509 (86.6%)	2.15 (1.39–3.32)**		

α Results are adjusted by the variables: Residence; Working; Husband's age.

β Results are adjusted by the variables: Age; Husband's age; Husband's education; Husband is older/younger.

γ Results are adjusted by the variables: Age; Husband is older/younger.

^*^p-value<0.05.

**p-value<0.01.

***p-value<0.001.

With regards to safe sex negotiation (Table 3), respondents were more likely to state in 2011: that they “Can refuse sexual intercourse” (OR 1.59, 95%, 1.49–1.71), that they “Can ask husband to use condom any time they want” (OR 1.90, 95%, 1.75–2.07), and that they were “Justified to ask husband to use condom if he has a Sexually Transmitted Disease (STD)” (OR 2.99, 95%, 2.76–3.25). Married women living in rural areas versus urban areas, and women married to a husband with lower education levels were significantly less likely to be able to negotiate safe sex. Higher education led to an increase in likelihood of between 1.3 and 7.5 times that a respondent agreed with the indicators. Higher socio-economic status and employment (except in the instance of “Can refuse sexual intercourse”) were also positively correlated with all safer sex negotiation indicators. Overall, women belonging to religious groups other than the Orthodox Christian faith had lower perceived power to negotiate safer sex. Respondents who had “Ever heard about AIDS” were significantly more likely to be able to negotiate safer sex but not the respondents who had “Ever heard of STIs”. An increase in age was only significantly associated with “Ever heard of AIDS”, and a husband's age or whether the husband was older/younger/same age as the respondent was not significantly correlated with any of these outcomes. Married women that were mothers were significantly less likely to feel “Justified to ask husband to use a condom if he has an STD” (OR 0.86, 95%, 0.74–0.99) but motherhood was not associated with the other indicators.

**Table 3:** Multinomial regression models showing factors significantly associated with empowerment to negotiate sex indicators.

Socio-Demographic Characteristics		Respondents N (%)	Can refuse sexual intercourseα	Can ask husband to use condom anytime she wantsα	Justified to ask husband to use a condom if he has an STD
			OR (95% CI)		
**Year**	2005	8,438 (47.1%)	ref.	ref.	ref.
	2011	9,478 (52.9%)	1.59 (1.49–1.71)***	1.90 (1.75–2.07)***	2.99 (2.76–3.25)***
**Residence**	Urban	3,719 (20.8%)	ref.	ref.	ref.
	Rural	14,197 (79.2%)	0.76 (0.67–0.87)***	0.61 (0.53–0.70)***	0.72 (0.62–0.85)***
**Education**	No education	12,545 (70.0%)	ref.	ref.	ref.
	Primary	3,716 (20.7%)	1.33 (1.21–1.45)***	1.84 (1.67–2.03)***	1.75 (1.58–1.95)***
	Secondary	1,201 (6.7%)	1.85 (1.55–2.21)***	4.18 (3.46–5.03)***	3.16 (2.48–4.03)***
	Higher	454 (2.5%)	2.15 (1.59–2.91)***	7.53 (5.19–10.93)***	4.32 (2.58–7.24)***
**Economic status**	Poorest	4,628 (25.8%)	ref.	ref.	ref.
	Poorer	3,057 (17.1%)	1.11 (1.01–1.23)*	1.42 (1.25–1.61)***	1.57 (1.40–1.77)***
	Middle	2,914 (16.3%)	1.11 (1.00–1.23)*	1.31 (1.15–1.50)***	1.73 (1.54–1.95)***
	Richer	2,873 (16.0%)	1.15 (1.04–1.28)**	1.45 (1.27–1.64)***	1.72 (1.52–1.93)***
	Richest	4,444 (24.8%)	1.48 (1.29–1.69)***	2.06 (1.76–2.40)***	2.77 (2.36–3.25)***
**Working**	No	12,789 (71.4%)	ref.	ref.	ref.
	Yes	5,112 (28.6%)	1.10 (1.03–1.19)**	1.25 (1.14–1.36)***	1.31 (1.19–1.43)***
**Religion**	Orthodox Christian	7,016 (39.2%)	ref.	ref.	ref.
	Muslim	7,360 (41.1%)	0.49 (0.45–0.52)***	0.64 (0.59–0.70)***	0.50 (0.45–0.54)***
	Other	360 (2.0%)	0.41 (0.31–0.52)***	0.76 (0.55–1.05)	0.53 (0.40–0.71)***
	Christian (Catholic & Protestant)	3,172 (17.7%)	0.42 (0.38–0.46)***	0.62 (0.55–0.69)***	0.51 (0.46–0.58)***
**Husband's education**	No education	9,723 (54.4%)	ref.	ref.	ref.
	Primary	5,293 (29.6%)	1.12 (1.03–1.21)**	1.24 (1.13–1.37)***	1.27 (1.16–1.40)***
	Secondary	1,903 (10.6%)	1.30 (1.13–1.49)***	1.54 (1.33–1.78)***	1.30 (1.10–1.53)**
	Higher	855 (4.8%)	1.51 (1.21–1.88)***	1.62 (1.28–2.03)***	1.52 (1.13–2.05)**
	Don't know	106 (0.6%)	1.97 (1.29–2.30)**	1.12 (0.70–1.80)	1.29 (0.79–2.10)
**Respondent is a mother**	No	1,792 (10.0%)			ref.
	Yes	16,124 (90.0%)			0.86 (0.74–0.99)*
**Heard of AIDS**	No	1,622 (9.1%)	ref.	ref.	ref.
	Yes	16,287 (90.9%)	1.98 (1.27–3.07)**	6.49 (2.35–17.91)***	5.73 (2.98–11.03)***

α Results are adjusted by the variable: Respondent is a mother.

*p-value<0.05.

**p-value<0.01.

***p-value<0.001.

## Discussion

The finding of most importance in this study is the demonstrated gender empowerment amongst married women between 2005 and 2011 in terms of knowledge of HIV and ability to negotiate safer sex. This suggests that the country's HIV programme is heading in the right direction. Some of these advancements were striking (e.g. “Heard of AIDS” improved by 3.6 times between 2005 and 2011 in the logistic regression model) and the question arises as to why these findings reflect such progress when a) Ethiopian HIV programmes largely target pre-married individuals (at the expense of older, married individuals), and b) most respondents do not have access to formal education. This progress may be partly due to Ethiopia's much-lauded community-based HIV sensitization programmes [20]. Indeed, the objectives of the Ethiopian Strategic Plan against HIV (2004–2008) highlight the emphasis placed on community sensitization: 1 of the 6 main thematic areas involves social mobilization and community empowerment [21] and the EDHS 2011 notes that community events have been critical in distributing information equally to all demographics [3]. That said, of concern is the observation that one of the six indicators of interest (“Having only one sex partner reduces the risk of HIV”) saw a drop over time – the reasons for this need to be explored and addressed.

Despite the advances achieved over time, the 2011 EDHS highlights the fact that married women consistently fared worse than their non-married counterparts (for instance: 62% vs. 77% agreed that they “Can ask husband to use a condom if he has an STD”; and 50% vs. 67% agreed that “Always using a condom reduces the risk of HIV”) [3]. These observations inevitably translate into a higher HIV prevalence rate for married (or cohabiting) women (1.5%) than for never-married women (0.5%) [3]. The challenges (including economic dependence and unequal gender roles) women face in a marriage have been quite extensively documented when negotiating safer sex [10]–[14]. Furthermore, the Ethiopian government has targeted much of its HIV programmes towards young and pre-married individuals through the education system and non-married women record considerably higher levels of education than married women (57% vs. 26% had attained primary education in 2011) [3]. Indeed, in this study an increase in education led to an increase of between 30% and about 9.5 times in HIV awareness or safer sex negotiation. The association between education and HIV knowledge has already been documented in multiple other studies [7], [22]–[24] and in Ethiopia the government has strongly supported HIV education in schools by incorporating HIV in the school curricula, by setting up HIV/AIDS clubs, and by encouraging school conversation programmes about HIV. The number of schools that organized these conversation programmes quadrupled from 3,255 to 15,305 between 2007/8 and 2012 [20].

Age was not significantly associated with safer sex negotiation or HIV awareness (except in the case of “Heard of AIDS”). One of the reasons may be that while an older respondent will have accumulated more knowledge about HIV and the importance of safe sex negotiation, the younger demographics will equally have begun to be informed about these topics through the education system and the various media outlets (radio, television, newspapers, etc.) that the government has significantly utilized to disseminate HIV information [3]. In addition, a husband's age and a husband's age relative to the respondent (older, same age, or younger) were not significantly associated with any of the indicators except in a couple of instances. The reasons for this need to be further studied although it may have been because women almost always had husbands older than they were. With regards to religion, women belonging to faiths other than Orthodox Christian had overwhelmingly lower HIV awareness and perceived power to negotiate safer sex. This may have been expected because historically, education in Ethiopia was distributed through the Orthodox Christian Church, and still today the national education language of instruction is Amharic – the official language of the Orthodox Church [25]. This may suggest that Orthodox Christian Ethiopians have greater access to education than respondents of other faiths. Indeed, Orthodox Christian believers attain Secondary or Higher education twice as much as the second largest religious group in the country [3]. Related to this is the observation that Orthodox Christian believers consistently have greater access to important sources of information such as the television and the radio when compared to believers of other faiths [3]. Also, the regions that are predominantly Muslim (such as Somali and Affar in the East) report the lowest rates of literacy in the country [3], which further highlights the importance of education in combating HIV/AIDS. Our results also reveal that a respondent's husband's level of education was a predictor, to some extent, of HIV awareness and was strongly associated with safer sex negotiation – perhaps because more educated males are less likely to engage in childhood marriage, marry much younger women, or, to encourage strict gender roles – factors strongly associated with HIV amongst women [16].

With the exception of one indicator, respondents that were employed were 10-30% more likely to be aware of HIV and to feel empowered to negotiate safer sex. This may be due to greater exposure to diverse environments and learning environments within the work setting as well as the financial or psychological independence that emboldens married women to face up to their partner. This finding was consistent with numerous other studies conducted on women, HIV, economic/social empowerment and safer sex negotiation [22]–[24]. This financial independence may also explain the reason why an increase in wealth was related to between an 11% and a 3.3-time increase in HIV awareness and safer sex negotiation with absolutely no exceptions. Increasing economic status has also been associated with increased access to mass media and thus information regarding sexual health [3], [26]. We should thus expect further improvement in these indicators as the Ethiopian economy powers ahead [1].

Urban living was associated with only one indicator pertaining to HIV awareness (“Always using a condom reduces risk of HIV”) and yet we assumed that urban living would have inferred greater exposure to information (notably through schools and media) translating into increased HIV awareness. However, this may be a good thing since it suggests that the HIV programmes are reaching urban populations as equally as rural ones. Ethiopia launched their acclaimed Health Extension Programme in 2003 and as of 2012, more than 30,000 health extension workers had been deployed to address disparities in delivering HIV prevention and health promotion services to rural areas [20]. As a result, when considering access to family planning messages, the EDHS 2011 reports disparities between urban and rural women in accessing medium such as television, radio, newspapers, posters or brochures but very little disparity in access to community-based education and behavioural change programmes (39.8% versus 35.7%) [3]. Nevertheless our analysis also reveals that married women in rural areas negotiate safer sex significantly less than women living in urban areas – HIV programmes need to address this disparity. This may be addressed by further expanding the community-based education and behavioural change programmes - reaching out to all sexually active people including youth about to initiate sexual activity. Furthermore, these women may feel more empowered to negotiate safer sex with the availability of employment opportunities afforded by the continued expansion of the national economy. Motherhood was not significantly associated with our selected indicators (except “Justified to ask husband to use a condom if he has an STD”) suggesting that the delivery of the HIV message during antenatal care and PMTCT services is falling short of expectations. The reasons for this need to be established and addressed since this service can be positively harnessed in tackling the HIV epidemic. Finally, as has been observed in other studies including one conducted in Ghana [24], respondents who had “Heard of HIV” were significantly more likely to be able to negotiate safer sex. Again, education in the form of HIV awareness positively impacted safer sex negotiation. However, respondents that had “Heard of STI” were not more likely to negotiate safer sex. Could this be because STIs are viewed by respondents as less harmful than HIV? The reasons for this difference need to be explored.

Overall this paper highlights the increased gender empowerment in Ethiopia's over time. However, as previously mentioned, the 2011 EDHS highlights the fact that married women fare worse than non-married women when it comes to negotiating safer sex [3]. Of further concern is the fact that the rates of HIV awareness are considerably lower than those seen in its neighbouring East-African countries: when it comes to identifying 2 methods of HIV prevention (restricting sexual contact to one uninfected partner and always using a condom) only 43% of Ethiopian women answered accurately in 2011 compared to 79% in Burundi (2010) [27], 71% in Kenya (2008–09) [28], 79% in Rwanda (2010) [29], 71% in Tanzania (2012) [30], and 66% in Uganda (2006) [31]. Evidently the reasons why Ethiopia lags behind its neighbours need to be studied but perhaps these differences can be explained by the much lower HIV prevalence rates (and thus urgency to educate) that the country has been faced with compared to her neighbours [27]–[31]. Ultimately, these differences between demographics (both within and outside Ethiopia) highlight the continued vulnerability of married Ethiopian women. Ethiopia has been lauded for its community sensitization programmes and these need to adapt to target the sub-groups of married women particularly vulnerable to HIV: the less educated, those not of the Orthodox Christian faith, those of lower socioeconomic standing or those that are not working, those who have never heard of HIV and, to some extent, those living in rural areas. Finally, the women's male spouses must not be ignored since they are the ones who are apparently responsible for increasing their wives' exposure to HIV. Promoting a change in behaviour is more likely to succeed if both men and women are informed of this need. Thus also targeting men through the aforementioned community sensitization programmes may prove useful. Messages would highlight the need to adjust their behaviour in order to benefit both their wife's health as well as well as their own (for instance by being encouraged to reduce the number of concurrent partners and to avoid unprotected sex). Messages could also promote openness to input from their wife when, for instance, they choose to negotiate safer sex conditions. This is particularly relevant since the 2011 EDHS highlights the fact that although the proportion of respondents who had two or more partners in the past 12 months was equally low amongst cohabiting and non-married women (0.3%), the proportion varied a lot more between non-married (1.2%) and married or cohabiting men (5.1%). Furthermore, the latter statistic appears to be rising since the proportion of married or cohabiting men who had two or more partners in 2005 was registered at 3.7%.

The quality of the dataset and the socio-demographic characteristics of the respondents are very reflective of the population of married women in Ethiopia, which allows us to draw apt conclusions. However, a major limitation this study faced is in the inherent setup of the DHS: respondents were only asked questions on safe sex negotiation regarding their immediate partner or husband. Had these questions been asked about their other concurrent partners, responses would presumably have varied as married women would have felt more empowered to negotiate safe sex with a non long-term partner whilst also giving pretexts such as not wanting to get pregnant outside of marriage. Either way, contrasting gender empowerment variables between the husband and another concurrent partner would have painted an interesting picture.

## Conclusions

The improvements observed between 2005 and 2011 mean that certain aspects of the Ethiopia's national HIV program are heading in the right direction. Education and economic empowerment have been strong contributing factors to these advancements. However, HIV programmes need to appreciate that married women are still disproportionately susceptible to HIV compared to men and unmarried women. Community programmes, already effective in Ethiopia, need to also target the sub-groups of married women most vulnerable: the less educated, those not of the Orthodox Christian faith, those of lower socioeconomic standing, those who have never heard of HIV/STIs and, to some extent, those living in rural areas.
